# Supplementary material for: A Critical View of the Application of the APEX Software (Aqueous Photochemistry of Environmentally-Occurring Xenobiotics) to Predict Photoreaction Kinetics in Surface Freshwaters
Source: Molecules. 2019 Dec 18;25(1):9. doi: 10.3390/molecules25010009 (PMC7017383; doi:10.3390/molecules25010009)
Supplement: Supplementary file 1 [file molecules-25-00009-s001.zip › 3.2.4_gcc-4.4.0.zip!/3.2.4_gcc-4.4.0/3.2.4_gcc-4.4.0/doc/octave/html/interpreter/Default-Arguments.html]

Default Arguments - Untitled


Next: Function Files,
Previous: Returning From a Function,
Up: Functions and Scripts


---

### 11.6 Default Arguments

Since Octave supports variable number of input arguments, it is very useful
to assign default values to some input arguments. When an input argument
is declared in the argument list it is possible to assign a default
value to the argument like this

```
     function name (arg1 = val1, ...)
       body
     endfunction
```

If no value is assigned to arg1 by the user, it will have the
value val1.

As an example, the following function implements a variant of the classic
“Hello, World” program.

```
     function hello (who = "World")
       printf ("Hello, %s!\n", who);
     endfunction
```

When called without an input argument the function prints the following

```
     hello ();
          -| Hello, World!
```

and when it's called with an input argument it prints the following

```
     hello ("Beautiful World of Free Software");
          -| Hello, Beautiful World of Free Software!
```

Sometimes it is useful to explicitly tell Octave to use the default value
of an input argument. This can be done writing a : as the value
of the input argument when calling the function.

```
     hello (:);
          -| Hello, World!
```
